# 

**Published:** 1898-10

**Authors:** 


### CONTENTS:

### SELECTIONS.

Article I.—Cardinal Points in the Successful Insertion of Permanent Fillings. Dr. H. C. Kahlo .................... 241
" II.—The Therapeutic Treatment of Infected Tooth Root-Canals. Dr. G. W. Warren .......................... 246
" III.—Tinfoil Matrices. W. Booth Pearson, F. R. C. S ..... 252
" IV.—Fees, and How to Collect them. Dr. A. B. Freeman .. 254
" V.—The Relation of the Teeth to the Lips and Face Dr. A. O. Hunt .......................................... 258
" VI.—Success. Dr. Geo. J. Manson ....................... 264
" VII.—A Few Dental Points. Dr. F. Mackinzie ............. 269
" VIII.—To Duplicate Models and Impressions. J. G. Templeton ...................................... 271
" IX.—Experiments With Alloys. Dr. J. D. Patterson ....... 276

### OBITUARY.

Dr. Gardner Quincy Colton ................................. 278

### MONTHLY SUMMARY.

How to Succeed ........................................... 279

Hardened and Washable Plaster-of-Paris .................... 280

Death Rate of Dentists .................................... 281

To Remove Enamel Previous to Crowning ..................... 282

Tempering Instruments ..................................... 283

One Way to Make a Gold Crown .............................. 284

Filling the Upper Bicuspids ............................... 285

Model for Partials ........................................ 286

Cement .................................................... 286

Pyorrhea .................................................. 287

Rinsing the Mouth ......................................... 287

Carious Dentine ........................................... 288

Amalgam Stain ............................................. 288

Oil of Cinnamon ........................................... 288

